# Development of a medical device compatible with MRI/CT to measure ankle joint laxity: the Porto Ankle Testing Device

**DOI:** 10.1097/j.pbj.0000000000000122

**Published:** 2021-02-11

**Authors:** Renato Andrade, Rogério Pereira, Ana Leal, Bruno Pereira, João Paulo Vilas Boas, C. Niek van Dijk, João Espregueira-Mendes

**Affiliations:** a Clínica do Dragão, Espregueira-Mendes Sports Centre—FIFA Medical Centre of Excellence, Porto, Portugal; Dom Henrique Research Centre, Porto, Portugal; Faculty of Sports, University of Porto; b Clínica do Dragão, Espregueira-Mendes Sports Centre—FIFA Medical Centre of Excellence, Porto, Portugal; Dom Henrique Research Centre, Porto, Portugal; Faculty of Sports, University of Porto, Portugal; Faculty of Health Sciences, University Fernando Pessoa, Porto; c CMEMS—Center for Micro-ElectroMechanical Systems, University of Minho, Campus Azurém, Guimarães; d Clínica do Dragão, Espregueira-Mendes Sports Centre—FIFA Medical Centre of Excellence, Porto, Portugal; Dom Henrique Research Centre, Porto, Portugal; Hospital Privado de Braga, Lugar da Igreja Nogueira, Braga; e Faculty of Sport, CIFI2D and Porto Biomechanics Laboratory (LABIOMEP), University of Porto, Porto, Portugal; f Clínica Do Dragão, Espregueira-Mendes Sports Center, FIFA Medical Centre of Excellence, Porto, Portugal; Department Orthopedic Surgery, Academic Medical Center (AMC), Amsterdam, The Netherlands; FIFA Medical Centre of Excellence Ripoll De Prado and Van Dijk Sport Clinic, Madrid, Spain; g Clínica do Dragão, Espregueira-Mendes Sports Centre—FIFA Medical Centre of Excellence, Porto, Portugal; Dom Henrique Research Centre, Porto, Portugal; School of Medicine, University of Minho, Braga, Portugal; ICVS/3B's-PT Government Associate Laboratory, Braga/Guimarães, Portugal; 3B's Research Group—Biomaterials, Biodegradables and Biomimetics, University of Minho, Headquarters of the European Institute of Excellence on Tissue Engineering and Regenerative Medicine, Barco, Guimarães, Portugal.

**Keywords:** ankle ligaments, diagnosis, joint laxity, magnetic resonance imaging

## Abstract

Ankle sprains are common and often develop into chronic ankle instability. Ankle laxity is usually assessed by manual testing followed by magnetic resonance imaging to confirm the diagnosis. Manual testing however provides a subjective measure and is limited to the assessor sensibility. Current available technologies incorporate arthrometers to objectively measuring ankle laxity, but are not capable to assess the structural integrity of the capsuloligamentous structures. To overcome these limitations, we developed a novel medical device to assist in the diagnosis of ankle ligament injuries—the Porto Ankle Testing Device. With this device, it is possible to combine and correlate the assessment of the capsuloligamentous’ structural integrity with the joint functional competence (ie, joint multiplanar laxity). The main purpose of this work is to present the fundamental aspects and step-by-step development of the Porto Ankle Testing Device. We discuss the design specifications and technical requirements with the purpose to design and develop this medical device, described the features of the different components and explained the mechanical systems that are incorporated emulate manual testing and to measure the multiplanar ankle laxity. The preliminary findings are presented with the purpose to display the assessment protocol, the method of laxity measurement and the obtained results. We propose a unique and reliable medical device to safety and effectively assess ankle ligament injuries and contribute to enhance diagnosis, refine treatment indications and allow objective measurement of ligament laxity before and/or after stabilization surgery.

To the Editor

We report the fundamental aspects, step-by-step development and preliminary validation in the relevant clinical context of a novel medical device that is compatible with magnetic resonance imaging (MRI) for the measurement of ankle joint laxity—the Porto Ankle Testing Device (PATD). We believe that the PATD can reliably and effectively improve ankle ligament injuries assessment and contribute to enhance diagnosis, refine treatment indications and allow objective measurement of ligament laxity before and/or after stabilization surgery.

## Introduction

Ankle sprains have a high incidence in athletes,^1–3^ but also among general population.^4^ These represent the most frequent musculoskeletal trauma of patients attending emergency departments,^3,5,6^ and yield therefore a high socio-economic impact.^3,7–12^ Recurrent sprains are also common and often develop into chronic ankle instability.^4,13–16^ Chronic ankle instability can result in cartilage lesions of the talus and early ankle osteoarthritis,^17–19^ implying an even higher socioeconomic costs. An early diagnosis of ankle laxity is thus crucial to prevent the development to chronic ankle instability and early osteoarthritis as well as to decrease the need for further intervention and associated costs.

The diagnosis of ankle instability is mostly determined by clinical history and physical exam performed by the clinician.^20^ The intra and inter-assessor reliability of physical tests is limited by experience and skill of the evaluator as well as by the sensibility and specificity of the tests employed.^21–23^ Several factors can affect the diagnostic accuracy of manual testing, including hand position and forces applied, ankle joint congruency, tissue stiffness variability, perception of movement and scoring methods.^22^ These divergencies in manual examination often lead to misdiagnosed ligament tears^24^ and underdiagnosed joint laxity.

The diagnosis is complemented by MRI assessment to evaluate the integrity of the ankle ligaments.^25,26^ However, the MRI is a static examination and does not assess the dynamic component of the joint that is directly related to the functional competence of the passive stabilization structures. In a chronic situation, the MRI is only capable of determining a change in the original structure which is interpreted as an old rupture—the MRI cannot tell us the amount of pathologic laxity. Ultrasound imaging has high reliability in assessing chronic ligament injuries,^27^ but is user-dependent, has lower imaging resolution and tissue depth, poor repeatability, and does not allow to standardize the application of joint stress. To overcome these drawbacks, several arthrometers have been developed to assess joint laxity. Stress radiography using the Telos™ device^28–34^ is commonly applied to assess joint displacement after stress, but involves exposure of the patient to radiation and does not allow the visualization of capsuloligamentous structures. Other arthrometers have been developed that do not require concomitant radiographic evaluation, such as the Ligmaster™,^35–39^ the quasi static anterior ankle tester (QAAT),^31,40^ the dynamic anterior ankle tester (DAAT),^31,41,42^ the Holis (Blue Bay Research Inc) arthrometer,^32,43–45^ among others.^46,47^ Evaluation of joint laxity or ligament stiffness (force-displacement curves) is made using electronic sensors that provides a gross estimate of joint displacement. All these devices have ferromagnetic materials incorporated, which makes them unsafe and incompatible within an MRI environment. These characteristics do not allow the visualization of bone and soft-tissue structures, which makes them uncapable to assess the structural integrity of capsuloligamentous structures and to objectively assess the true joint displacement (ie, joint laxity). Aiming to overcome these limitations we developed a medical device that allows to objectively measure ankle laxity (functional competence) while allowing to evaluate the status of capsuloligamentous structures (structural integrity).

## Design specifications and technical requirements

The medical device was designed to measure the ankle laxity, that is, the multiplanar displacement after the application of an external mechanical force. The PATD emerges from a research and development line of medical devices to measure joint laxity (patent number: US10470700), including the Porto Knee Testing Device (PKTD^®^)^48–53^ and the Porto Patella Testing Device (PPTD).^54–56^

**Figure 1:**
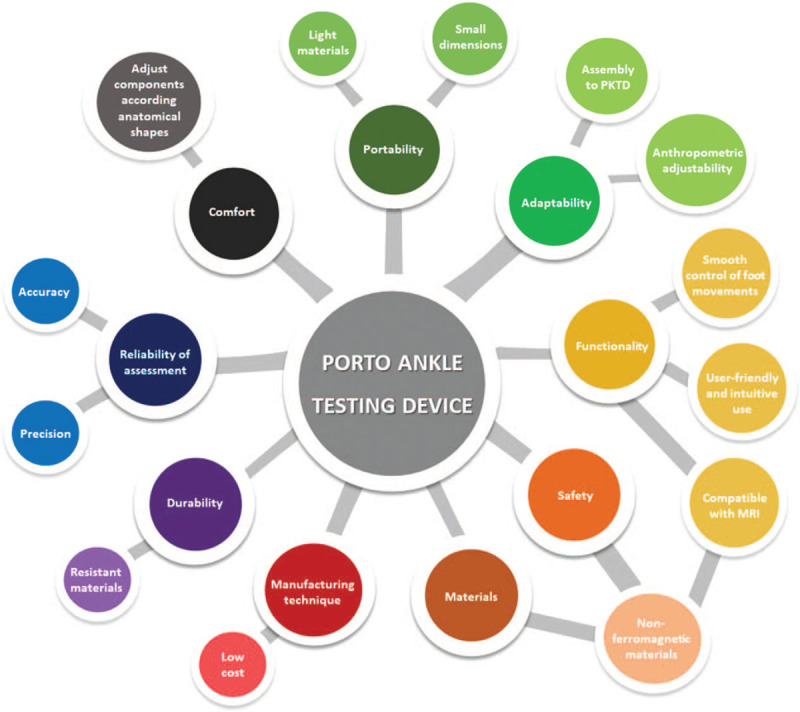
Design specifications and technical requirements for the development of the PATD.

The PATD has to combine several *design specifications* and *technical requirements*—that are inherent to medical devices—to ensure safety and optimal performance, as well as to allow to reliably assess ankle laxity (Fig. 1). The most relevant *technical requirements* that are specific for the PATD are further explained bellow:

i. Functional capacity: the medical device needs to emulate the manual tests (anterior drawer and valgus/varus stress) that are performed during the physical exam by the application of an external force.
  (a) Anterior drawer test: posteroanterior stress at the posterior aspect of the calcaneus with fixation of the distal tibia and peroneus;
  (b) Valgus/varus test: eversion/inversion stress at the medial/lateral aspect of the calcaneus with fixation at the instep of the foot.
ii. Compatibility and safety within imaging environment: MRI imaging is required to determine the structural status of the ligament. Combining the MRI and laxity assessment allows to correlate the structural integrity with the functional competence (ie, joint laxity) of the capsuloligamentous structures. For measuring joint laxity, it is recorded the resting position and calculated the talocrural and subtalar displacements after stress, for which it is required to visualize the bony structures. To ensure compatibility and safety within the MRI environment, the medical device must be free of ferromagnetic materials and has to fit the maximum volume of a standard MRI machine (500 × 400 × 400 mm).
iii. Compatibility and adaptability to PKTD^®^: the PATD module needs to be compatible and able to couple with the PKTD^®^ main structure. The PKTD^®^ will act as a support for the lower limb and allow to adjust knee flexion angle.
iv. Ergonomic and anthropometric adjustability: the device must provide anatomical comfort to the patient and offer a user-friendly solution for the device operator. The medical device must allow to evaluate both left and right ankles and consider anthropometric adjustment according to individual variability, including leg and foot length.

## Prototype development

**Figure 2:**
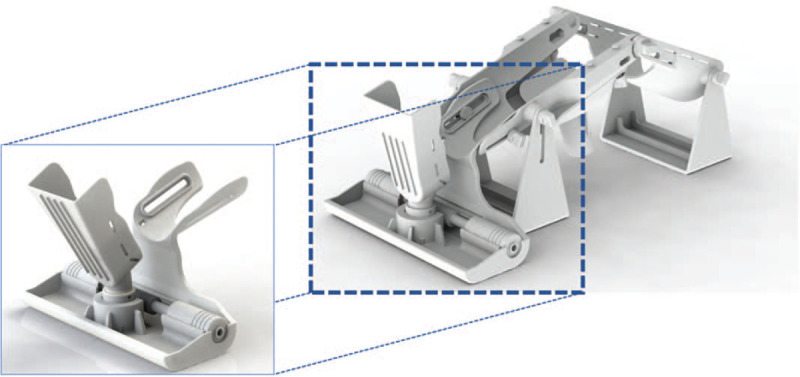
Full prototype of the PATD (image on the left) and linkage to the existing PKTD (image on the right).

The development of a PATD prototype considered 3 main characteristics: assembly to the PKTD, foot support and fixation, and mechanical components for multiplanar movement. A computer-aided-design model was developed using SolidWorks software to simulate the iterations of the desired movements and material fatigue tests for the final version of PATD prototype—depicted in Figure 2.

### Assembly to PKTD^®^

The PATD module was designed to be assembled to the existing medical device PKTD^®^, which will serve as support for the lower limb and to control the knee flexion angle. The geometry of the linkage between the PATD and PKTD^®^ must be designed to adapt and fit the distal module of the PKTD^®^ (Fig. 2). The linkage part is designed to adjust to the leg length of each individual. At the distal lateral and medial borders of the linked support structure, there is a pair of slots where a Velcro strap will be tightened to block the distal tibia against posteroanterior movement.

### Foot platform and fixation

**Figure 3:**
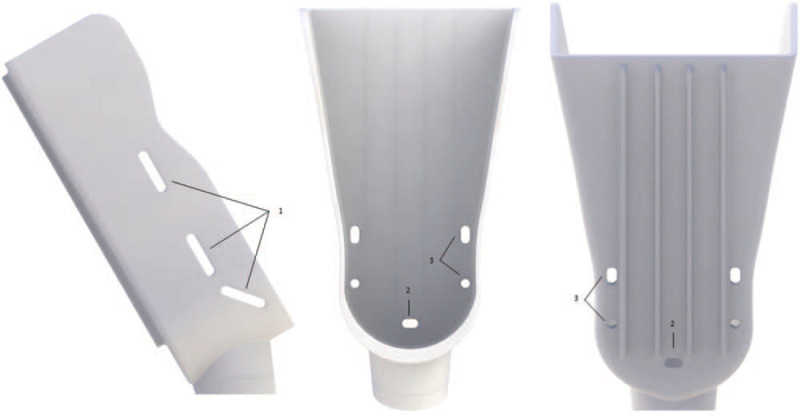
Foot platform of the PATD, from the lateral, anterior and posterior perspectives. (1) Bilateral slots where Velcro straps are fastened for foot fixation; (2) inferior; (3) and side pin fitting for where adjustable foot base which is used for valgus/varus stress test.

The foot platform (Fig. 3) was developed using a standard anatomical shape to fit a wide range of different foot dimensions. Around the medial and lateral malleolus, the foot platform was molded to prevent conflict with the malleolus bony structure and thus avoid pain by contact or compression. The platform contains 3 pairs of slots—1 posterior and 2 intermediates—where Velcro straps will be fastened during laxity testing to fixate the foot to the platform and avoid undesired foot movement. The posterior wall of the foot platform is reinforced by 4 longitudinal strips to dissipate forces throughout the platform and thus avoid accumulated torsion tensions at a single point, preventing material breakage. The platform is inclined forward placing the foot at 15 degrees of plantar flexion to unlock the talus from the ankle mortise and allow unrestricted tibiotalar movement.

### Anterior drawer mechanical system

**Figure 4:**
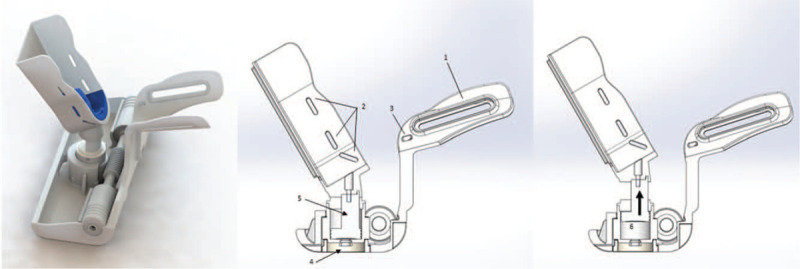
PATD mechanism to emulate the anterior drawer test. The pneumatic system (arrow in the image on the right) is actuated through bariatric pressure (up to 4.0 Bar) that applies a posteroanterior stress at the calcaneus. (1) Linkage structure of the PATD where it assembles to the PKTD structure; (2) and (3) bilateral slots where Velcro straps are fastened for foot (2) and distal leg (3) fixation; (4) inferior connector where the air hose hooks for posteroanterior stress; (5) pneumatic cylinder; (6) air is pumped through the connector pushing the piston within the pneumatic cylinder upwards (as shown by arrow).

To emulate the anterior drawer test of the ankle joint, the mechanical system needs to apply a posteroanterior stress at the heel while the lower tibia is fixed anteriorly to restrict the posteroanterior movement to the talus and calcaneus. The stress is exerted through the foot platform (the foot platform moves anteriorly) actuated by a nonmagnetic pneumatic cylinder that is triggered by bariatric pressure through air pumping (Fig. 4). The bariatric pressure can go up to 4.0 Bar, but the anterior drawer mechanism is performed until the patient's pain threshold.

#### Anterolateral or anteromedial laxity testing foot base

**Figure 5:**
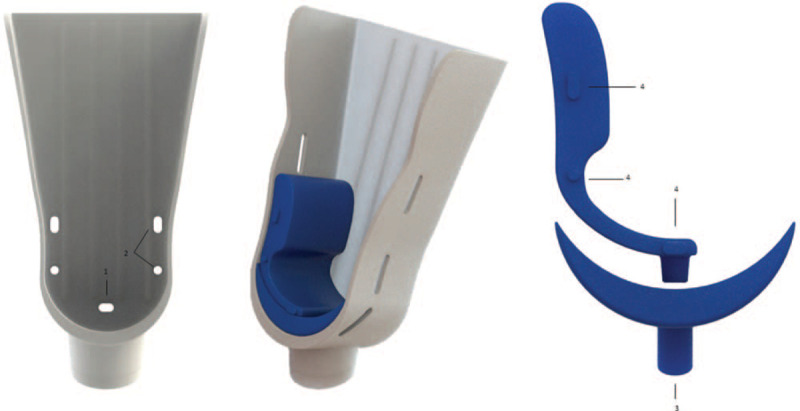
Adjustable foot base to enforce an internal or external rotation. The foot base has 2 components that fit in notches at the foot platform. (1) Inferior and (2) side pin fitting for the adjustable foot base; (3) inferior silicon pin that fits into the foot platform; (4) inferior silicon pin that fits on top of the inferior component of the adjustable foot base and lateral silicon pins that fit in the lateral borders of foot platform.

To measure the anterolateral or anteromedial laxity, a base piece (Fig. 5) was developed to be assembled at the foot platform through 4 sets of notches. The foot base piece is composed by silicone materials and divided into 2 modules. The first base module is placed at the bottom of the foot platform and provides comfort at the heel (where the stress will be applied). The second module is linked on the top of the inferior module and should be placed in the lateral or medial side to enforce an internal or external rotation to the ankle joint while performing the posteroanterior stress to allow to test the anterolateral or anteromedial rotational laxity testing, respectively.

### Valgus/varus stress test mechanical system

**Figure 6:**
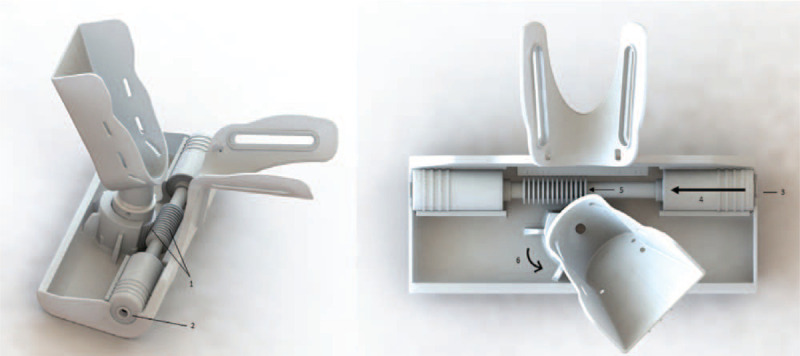
PATD mechanism to emulate the valgus/varus stress test. The pneumatic system (arrow in the image on the right) is actuated through bariatric pressure (up to 4.0 Bar) that applies a rotating stress at the foot. (1) Rack and pinion mechanism that activates the rotational movement; (2) lateral connector where the air hose hooks for inversion/eversion stress; (3) the air is pumped through the lateral connector; (4) which moves the pneumatic cylinder; (5) activate the rack and pinion mechanism to produce in inversion/eversion stress (6).

To emulate the valgus/varus stress test of the ankle joint, the mechanical system needs to apply an eversion/inversion stress at the medial/lateral aspect of the hindfoot and midfoot with the foot fixed at the instep. The eversion/inversion stress is applied through the foot platform (the foot platform rotates internal or externally). The platform rotational movement is actuated by a rack and pinion mechanism at the base of the platform through the use of pneumatic cylinders as described previously for the anterior drawer (Fig. 6). The bariatric pressure can go up to 4.0 Bar, but the anterior drawer mechanism is performed until the patient's pain threshold.

## Prototype fabrication

The final physical prototype was built using poly-based materials (resins and composites), free of any ferromagnetic chemical materials (eg, iron, nickel, cobalt) as well as electric or electronic components, because these components are not compatible and safe within MRI environment and cause image distortion due to the strong magnetic fields produced by the MRI. Using the final design model, a first prototype was built using stereothography additive technology. With this first prototype, it could be performed the functional and technical evaluation in cadaveric specimens and then in human subjects within an MRI environment, to test if all the PATD components were operating as desired. Following the validation of the functional and technical requirements, the final version of this medical device prototype was manufactured using polyurethane materials (PR 403, Synthene).

## Preliminary results of PATD-MRI evaluation

The PATD was tested within MRI environment in patients with chronic ankle instability using the protocol for anterolateral ankle laxity. Using the MRI visualization, it allows an objective measure of the joint position to calculate the displacement after stress, providing a more accurate and precise evaluation of ankle laxity and allowing a more rigorous diagnosis of mechanical ankle laxity.

**Figure 7:**
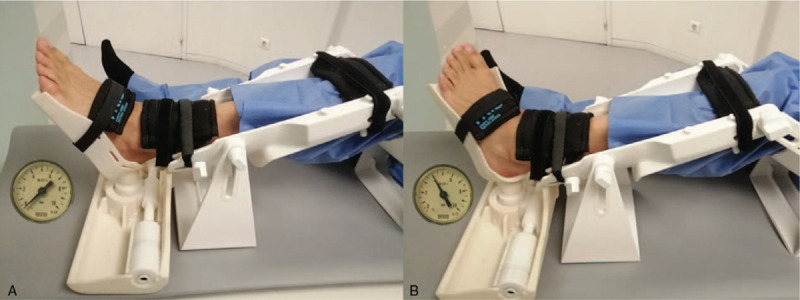
Lower limb and foot placement for PATD testing. (A) Placement for the rest condition and (B) after inversion stress (4.0 Bar). The barometer displays the bariatric pressure being applied.

**Figure 8:**
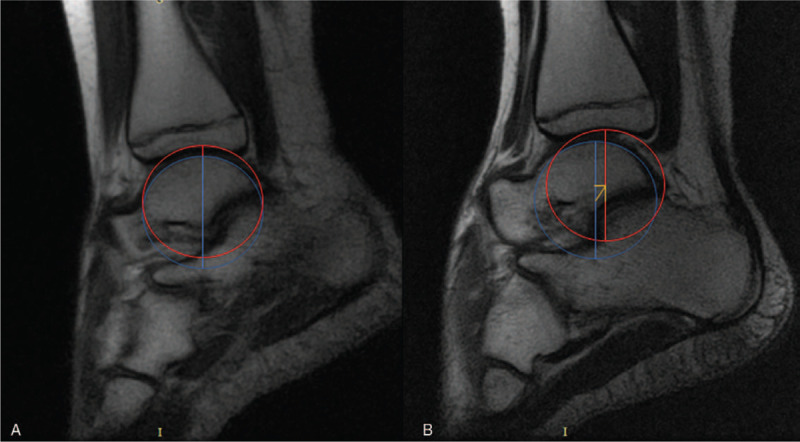
PATD assessment for anterior drawer test under MRI control. (A) Rest position and (B) after posteroanterior stress (the orange lines show the anterior, inferior and diagonal translation of the talus).

**Figure 9:**
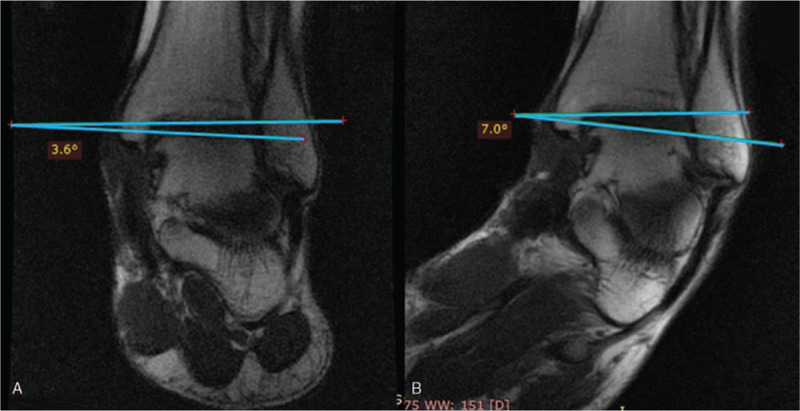
PATD assessment for varus stress test under MRI control. (A) Rest position and (B) after inversion stress (the blue lines show the angle between the distal surface of the tibia and the proximal surface of the talar dome).

A few patients with chronic ankle instability were evaluated to test and validate the device in the clinically relevant context using a standardized and reproducible PATD-MRI evaluation protocol that consisted in 3 steps. First, the lower limb is positioned in the PATD with the knee flexed at 50 degrees and Velcro straps are fasten at the thigh, upper and lower tibia, and at the foot instep. A first set of images (sagittal, coronal and axial views) is scanned with the joint in the rest position (Fig. 7A). Then, another set of images (sagittal view) is scanned while performing the anterior drawer and then another set (coronal view) after the inversion stress test (Fig. 7B). For the anterior drawer test, the talus anterior displacement is calculated by comparing the center talus position in relation to the distal tibial central axis with the ankle joint in rest and after posteroanterior stress. Figure 8 displays the MRI sagittal view of the ankle in rest (Fig. 8A) and after anterior translation (Fig. 8B). The posteroanterior stress moved the talus (in relation to the distal tibial surface) 7.2 mm in the anterior direction, 4.1 mm in the inferior direction and 8.3 mm in the diagonal (antero-inferior) direction. For the inversion test, the tibiotalar opening angle is calculated by comparing the position of the ankle joint in rest and after inversion stress. Figure 9 displays the MRI coronal view of the ankle in rest (Fig. 9A) and after inversion stress (Fig. 9B). The inversion opened the tibiotalar angle by 3.4 degrees. These results suggest the static stabilizers of the patient's ankle are incompetent, highlighted by the pathological sagittal laxity, and that may warrant treatment.

## Conclusion

The PATD offers a more accurate and precise evaluation of ankle laxity and a more rigorous diagnosis of mechanical ankle laxity. It is safe and compatible within MRI environment and thus allows to correlate the capsuloligamentous structural integrity with its functional competence. The preliminary testing in cadaver specimens and human subjects shows that the PATD is able to accurately measure bony displacement after stress with the aid of MRI scanning.

The PATD can be of great value for the diagnosis and treatment of sprained or chronically injured ankles, including objectively quantify ankle mechanical stability, refine treatment indications (conservative vs surgical interventions) and follow-up of conservative/surgical interventions.

## Acknowledgments

The authors would like to thank Joana Arantes Silva, Álvaro Araújo and Lino Abreu for their assistance in the design and development of this device.

## Conflicts of interest

JEM is the patent holder of US10470700 which includes parts of this medical device. He receives no royalties or fees.

## References

[1] Hootman JM Dick R Agel J. Epidemiology of collegiate injuries for 15 sports: summary and recommendations for injury prevention initiatives. J Athl Train. 2007;42:311–319. 17710181 PMC1941297

[2] Cameron KL Marshall SW Sturdivant RX Lincoln AE. Trends in the incidence of physician-diagnosed mild traumatic brain injury among active duty U.S. military personnel between 1997 and 2007. J Neurotrauma. 2012;29:1313–1321. 22332633 10.1089/neu.2011.2168

[3] Shah S Thomas AC Noone JM Blanchette CM Wikstrom EA. Incidence and cost of ankle sprains in United States emergency departments. Sports Health. 2016;8:547–552. 27474161 10.1177/1941738116659639 PMC5089353

[4] Hiller CE Nightingale EJ Raymond J Prevalence and impact of chronic musculoskeletal ankle disorders in the community. Arch Phys Med Rehabil. 2012;93:1801–1807. 22575395 10.1016/j.apmr.2012.04.023

[5] Fong DT Hong Y Chan LK Yung PS Chan KM. A systematic review on ankle injury and ankle sprain in sports. Sports Med. 2007;37:73–94. 17190537 10.2165/00007256-200737010-00006

[6] Fong DT Man CY Yung PS Cheung SY Chan KM. Sport-related ankle injuries attending an accident and emergency department. Injury. 2008;39:1222–1227. 18538772 10.1016/j.injury.2008.02.032

[7] Cooke MW Marsh JL Clark M Treatment of severe ankle sprain: a pragmatic randomised controlled trial comparing the clinical effectiveness and cost-effectiveness of three types of mechanical ankle support with tubular bandage. The CAST trial. Health Technol Assess. 2009;13:iii, ix–x, 1–121. 10.3310/hta13130 19232157

[8] Hupperets MD Verhagen EA Heymans MW Bosmans JE van Tulder MW van Mechelen W. Potential savings of a program to prevent ankle sprain recurrence: economic evaluation of a randomized controlled trial. Am J Sports Med. 2010;38:2194–2200. 20699429 10.1177/0363546510373470

[9] Janssen KW Hendriks MR van Mechelen W Verhagen E. The cost-effectiveness of measures to prevent recurrent ankle sprains: results of a 3-Arm randomized controlled trial. Am J Sports Med. 2014;42:1534–1541. 24753237 10.1177/0363546514529642

[10] Knowles SB Marshall SW Miller T Cost of injuries from a prospective cohort study of North Carolina high school athletes. Inj Prev. 2007;13:416–421. 18056320 10.1136/ip.2006.014720 PMC2598289

[11] Lin CW Uegaki K Coupe VM Kerkhoffs GM van Tulder MW. Economic evaluations of diagnostic tests, treatment and prevention for lateral ankle sprains: a systematic review. Br J Sports Med. 2013;47:1144–1149. 22554849 10.1136/bjsports-2012-090319

[12] Olmsted LC Vela LI Denegar CR Hertel J. Prophylactic ankle taping and bracing: a numbers-needed-to-treat and cost-benefit analysis. J Athl Train. 2004;39:95–100. 15085217 PMC385268

[13] Anandacoomarasamy A Barnsley L. Long term outcomes of inversion ankle injuries. Br J Sports Med. 2005;39:e14–e114. 15728682 10.1136/bjsm.2004.011676 PMC1725165

[14] Doherty C Bleakley C Hertel J Caulfield B Ryan J Delahunt E. Recovery from a first-time lateral ankle sprain and the predictors of chronic ankle instability: a prospective cohort analysis. Am J Sports Med. 2016;44:995–1003. 26912285 10.1177/0363546516628870

[15] Konradsen L Bech L Ehrenbjerg M Nickelsen T. Seven years follow-up after ankle inversion trauma. Scand J Med Sci Sports. 2002;12:129–135. 12135444 10.1034/j.1600-0838.2002.02104.x

[16] Swenson DM Yard EE Fields SK Comstock RD. Patterns of recurrent injuries among US high school athletes, 2005–2008. Am J Sports Med. 2009;37:1586–1593. 19372270 10.1177/0363546509332500

[17] Taga I Shino K Inoue M Nakata K Maeda A. Articular cartilage lesions in ankles with lateral ligament injury. An arthroscopic study. Am J Sports Med. 1993;21:120–126. discussion 126-127. 8427352 10.1177/036354659302100120

[18] Valderrabano V Horisberger M Russell I Dougall H Hintermann B. Etiology of ankle osteoarthritis. Clin Orthop Relat Res. 2009;467:1800–1806. 18830791 10.1007/s11999-008-0543-6 PMC2690733

[19] Valderrabano V Hintermann B Horisberger M Fung TS. Ligamentous posttraumatic ankle osteoarthritis. Am J Sports Med. 2006;34:612–620. 16303875 10.1177/0363546505281813

[20] Delahunt E Bleakley CM Bossard DS Clinical assessment of acute lateral ankle sprain injuries (ROAST): 2019 consensus statement and recommendations of the International Ankle Consortium. Br J Sports Med. 2018;52:1304–1310. 29886432 10.1136/bjsports-2017-098885

[21] Van Dijk CN Mol BWJ Lim LS Marti RK Bossuyt PM. Diagnosis of ligament rupture of the ankle joint: physical examination, arthrography, stress radiography and sonography compared in 160 patients after inversion trauma. Acta Orthop Scand. 1996;67:566–570. 9065068 10.3109/17453679608997757

[22] Fujii T Luo Z-P Kitaoka HB An K-N. The manual stress test may not be sufficient to differentiate ankle ligament injuries. Clin Biomech. 2000;15:619–623. 10.1016/s0268-0033(00)00020-6 10936435

[23] Van Dijk C. On Diagnostic Strategies in Patients with Severe Ankle Sprain (Thesis). The Netherlands: University of Amsterdam; 1994.

[24] Lähde S Putkonen M Puranen J Raatikainen T. Examination of the sprained ankle: anterior drawer test or arthrography? Eur J Radiol. 1988;8:255–257. 3148464

[25] Kim YS Kim YB Kim TG Reliability and validity of magnetic resonance imaging for the evaluation of the anterior talofibular ligament in patients undergoing ankle arthroscopy. Arthroscopy. 2015;31:1540–1547. 25882180 10.1016/j.arthro.2015.02.024

[26] Takao M Innami K Matsushita T Uchio Y Ochi M. Arthroscopic and magnetic resonance image appearance and reconstruction of the anterior talofibular ligament in cases of apparent functional ankle instability. AM J Sports Med. 2008;36:1542–1547. 18443275 10.1177/0363546508315537

[27] Cao S Wang C Ma X Wang X Huang J Zhang C. Imaging diagnosis for chronic lateral ankle ligament injury: a systemic review with meta-analysis. J Orthop Surg Res. 2018;13:122–1122. 29788978 10.1186/s13018-018-0811-4 PMC5964890

[28] Karlsson J Bergsten T Lansinger O Peterson L. Reconstruction of the lateral ligaments of the ankle for chronic lateral instability. J Bone Joint Surg Am. 1988;70:581–588. 3356725

[29] Jung HG Kim NR Kim TH Eom JS Lee DO. Magnetic resonance imaging and stress radiography in chronic lateral ankle instability. Foot Ankle Int. 2017;38:621–626. 28552041 10.1177/1071100717693207

[30] Lee KT Park YU Jegal H Park JW Choi JP Kim JS. New method of diagnosis for chronic ankle instability: comparison of manual anterior drawer test, stress radiography and stress ultrasound. Knee Surg Sports Traumatol Arthrosc. 2014;22:1701–1707. 24067992 10.1007/s00167-013-2690-x

[31] Kerkhoffs GM Blankevoort L Sierevelt IN Corvelein R Janssen GH van Dijk CN. Two ankle joint laxity testers: reliability and validity. Knee Surg Sports Traumatol Arthrosc. 2005;13:699–705. 16028054 10.1007/s00167-005-0644-7

[32] Hubbard TJ Kaminski TW Vander Griend RA Kovaleski JE. Quantitative assessment of mechanical laxity in the functionally unstable ankle. Med Sci Sports Exerc. 2004;36:760–766. 15126707 10.1249/01.mss.0000126604.85429.29

[33] Beynnon BD Webb G Huber BM Pappas CN Renstrom P Haugh LD. Radiographic measurement of anterior talar translation in the ankle: determination of the most reliable method. Clin Biomech (Bristol, Avon). 2005;20:301–306. 10.1016/j.clinbiomech.2004.11.011 15698703

[34] Nyska M Amir H Porath A Dekel S. Radiological assessment of a modified anterior drawer test of the ankle. Foot Ankle. 1992;13:400–403. 1427531 10.1177/107110079201300707

[35] Rosen A Ko J Brown C. A multivariate assessment of clinical contributions to the severity of perceived dysfunction measured by the Cumberland Ankle Instability Tool. Int J Sports Med. 2016;37:1154–1158. 27706549 10.1055/s-0042-113464

[36] Rosen AB Ko J Brown CN. Diagnostic accuracy of instrumented and manual talar tilt tests in chronic ankle instability populations. Scand J Med Sci Sports. 2015;25:e214–e221. 24995627 10.1111/sms.12288

[37] Wikstrom EA Tillman MD Chmielewski TL Cauraugh JH Naugle KE Borsa PA. Dynamic postural control but not mechanical stability differs among those with and without chronic ankle instability. Scand J Med Sci Sports. 2010;20:e137–e144. 19422654 10.1111/j.1600-0838.2009.00929.x

[38] Hirai D Docherty CL Schrader J. Severity of functional and mechanical ankle instability in an active population. Foot Ankle Int. 2009;30:1071–1077. 19912717 10.3113/FAI.2009.1071

[39] Docherty CL Rybak-Webb K. Reliability of the anterior drawer and talar tilt tests using the LigMaster joint arthrometer. J Sport Rehabil. 2009;18:389–397. 19827502 10.1123/jsr.18.3.389

[40] Kerkhoffs GMMJ Blankevoort L van Dijk CN. A measurement device for anterior laxity of the ankle joint complex. Clin Biomech. 2005;20:218–222. 10.1016/j.clinbiomech.2004.09.006 15621328

[41] de Vries JS Kerkhoffs GMMJ Blankevoort L van Dijk CN. Clinical evaluation of a dynamic test for lateral ankle ligament laxity. Knee Surg Sports Traumatol Arthrosc. 2010;18:628–633. 19924401 10.1007/s00167-009-0978-7 PMC2855027

[42] Kerkhoffs GMMJ Blankevoort L Schreurs AW Jaspers JEN van Dijk CN. An instrumented, dynamic test for anterior laxity of the ankle joint complex. J Biomech. 2002;35:1665–1670. 12445620 10.1016/s0021-9290(02)00189-6

[43] Terada M Bowker S Hiller CE Thomas AC Pietrosimone B Gribble PA. Quantifying levels of function between different subgroups of chronic ankle instability. Scand J Med Sci Sports. 2017;27:650–660. 27292532 10.1111/sms.12712

[44] Hubbard-Turner T Turner MJ. Physical activity levels in college students with chronic ankle instability. J Athl Train. 2015;50:742–747. 25898110 10.4085/1062-6050-50.3.05 PMC4532186

[45] Hubbard-Turner T. Relationship between mechanical ankle joint laxity and subjective function. Foot Ankle Int. 2012;33:852–856. 23050709 10.3113/FAI.2012.0852

[46] Liu W Siegler S Techner L. Quantitative measurement of ankle passive flexibility using an arthrometer on sprained ankles. Clin Biomech (Bristol, Avon). 2001;16:237–244. 10.1016/s0268-0033(00)00088-7 11240059

[47] Dressler P Gehring D Zdzieblik D Oesser S Gollhofer A Konig D. Improvement of functional ankle properties following supplementation with specific collagen peptides in athletes with chronic ankle instability. J Sports Sci Med. 2018;17:298–304. 29769831 PMC5950747

[48] Espregueira-Mendes J Pereira H Sevivas N Assessment of rotatory laxity in anterior cruciate ligament-deficient knees using magnetic resonance imaging with Porto-knee testing device. Knee Surg Sports Traumatol Arthrosc. 2012;20:671–678. 22290127 10.1007/s00167-012-1914-9

[49] Espregueira-Mendes J Andrade R Leal A Global rotation has high sensitivity in ACL lesions within stress MRI. Knee Surg Sports Traumatol Arthrosc. 2017;25:2993–3003. 27530386 10.1007/s00167-016-4281-0

[50] Bastos R Andrade R Vasta S Tibiofemoral bone bruise volume is not associated with meniscal injury and knee laxity in patients with anterior cruciate ligament rupture. Knee Surg Sports Traumatol Arthrosc. 2019;27:3318–3326. 30604253 10.1007/s00167-018-5343-2

[51] Pereira H Gomes S Vasconcelos JC. Musahl V Karlsson J Kuroda R Zaffagnini S MRI laxity assessment. Rotatory Knee Instability. Berlin, Heidelberg: Springer; 2017;49–61.

[52] Andrade R Pereira R Bastos R. Musahl V Karlsson J Krutsch W Mandelbaum BR Espregueira-Mendes J d’Hooghe P MRI-based laxity measurement for return to play. Return to Play in Football: An Evidence-based Approach. Berlin, Heidelberg: Springer; 2018;205–215.

[53] Andrade R Duarte H Pereira R. Margheritini F Espregueira-Mendes J Gobbi A An advanced device for multiplanar instability assessment in MRI. Complex Knee Ligament Injuries. Berlin, Heidelberg: Springer; 2019;27–33.

[54] Leal A Andrade R Flores P Unilateral anterior knee pain is associated with increased patellar lateral position after stressed lateral translation. Knee Surg Sports Traumatol Arthrosc. 2020;28:454–462. 31375878 10.1007/s00167-019-05652-7

[55] Leal A Andrade R Hinckel B Patients with different patellofemoral disorders display a distinct ligament stiffness pattern under instrumented stress testing. J ISAKOS. 2020;5:74–79.

[56] Leal A Andrade R Hinckel BB A new device for patellofemoral instrumented stress-testing provides good reliability and validity. Knee Surg Sports Traumatol Arthrosc. 2020;28:389–397. 31250058 10.1007/s00167-019-05601-4

